# Clinicopathological characteristics and molecular abnormalities of primary grade 2 neuroendocrine tumors of the cervix

**DOI:** 10.1186/s13000-019-0837-x

**Published:** 2019-06-22

**Authors:** Ran Zhu, Huanwen Wu, Bo Chen, Junyi Pang, Zhen Huo

**Affiliations:** 1 Department of Pathology, Peking Union Medical College Hospital, Chinese Academy of Medical Sciences & Peking Union Medical College, No.1Shuaifuyuan, Wangfujing Street, Dongcheng District, Beijing, 100730 China; 2 Department of Pathology, Changping Hospital of Integrated Chinese and Western Medicine, Beijing, 102208 China

**Keywords:** Primary grade 2 neuroendocrine tumors, Cervix, Clinicopathological characteristics, Molecular abnormalities, Immunocytochemistry

## Abstract

**Background:**

Primary grade 2 neuroendocrine tumors of the cervix in female patients are rare and have a highly aggressive clinical course. This study is aimed to analyse the diagnosis, genetic changes, management and prognosis of these tumors and investigate whether the genetic alterations could provide more useful information to guide the molecular characterization and potential individualized treatment of grade 2 cervical neuroendocrine tumors.

**Methods:**

The clinical records of all three patients diagnosed as primary grade 2 neuroendocrine tumors of the cervix in Peking Union Medical College Hospital (PUMCH) from 2011 to 2018 were reviewed retrospectively. We investigated the morphology, immunophenotype and molecular abnormalities of all the cases. The follow-up data were also collected.

**Results:**

The age of the patients ranged from 46 to 69 years. All cases were in stage II and treated with surgery. The microscopic examination showed that the tumors took the form of nest-like, trabecular, sheet-like, “single file” strands or rosette-like structures. The mitotic figures ranged from 2 to 5 in every 10 high-power fields, and necrotic foci were observed in one case. Immunohistochemically, the tumor cells were positive for AE1/AE3, Cg A, Syn, CD56, P16, CAM5.2, and PGP9.5 and negative for ER, PR, P63, P40, CK7, and CK20. The expression of P53 showed as normal/wild-type pattern, and the proliferation index of Ki-67 ranged from 2 to 7%. A total of 560 genes were sequenced by next-generation sequencing for each patient, and nonsynonymous somatic mutations were identified in the three cases. Non-frameshift insertions of the MAGI1 and SLC45A were both observed in case 1, while we only observed the non-frameshift insertion of the MAGI1 in case 2 and the non-frameshift insertion of the SLC45A in case 3. Case 1 was treated with chemoradiotherapy before and after surgery. Cases 2 and 3 were treated with chemotherapy before and after surgery. The follow-up time ranged from 27 to 74 months. Cases 2 and 3 survived, while case 1 died.

**Conclusion:**

Cervical grade 2 neuroendocrine tumors are extremely rare. We presented the first mutation profile revealed by whole exome sequencing in a series of grade 2 cervical NETs along with their clinicopathological characteristics. Their genetic changes are different from those that take place in the gastrointestinal tract, pancreas and lung, which have gene changes in VEGF, RTKs or the mTOR signalling pathway. While changes in MAGI1 and SLC45L3 were observed in two of our cases and the case who had the gene changes of both MAGI1 and SLC45L3 died because of metastases to the liver and bone. The genetic alterations may provide more useful information to guide the molecular characterization and potential individualized treatment of grade 2 cervical neuroendocrine tumors.

## Background

Neuroendocrine tumors (NETs) are heterogeneous tumors showing diverse clinical and biological characteristics that predominantly occur in the gastrointestinal tract, pancreas and lung, but are rarely found in other locations of the body. NETs of the uterine cervix are rare, accounting for 0.9 to 1.5% of cervical malignancies [1].

According to the updated 2014 World Health Organization (WHO) classification of tumors [2], cervical NETs are categorized as low-grade NETs (including grade 1 and grade 2) and high-grade NETs (including small cell neuroendocrine carcinoma and large cell neuroendocrine carcinoma). Grade 2 NETs are exceedingly rare such that only 17 cases have been documented in the past two decades [3–6]. Additionally, they are considered to be highly aggressive tumors that usually feature lympho-vascular space invasion and lymph node involvement [3–6]. Accordingly, no guidelines on the treatment of these tumors are available at present. Currently, biologic-targeted therapies derived from engineered gene products play a particularly important role in the treatment of well-differentiated NETs occurring in the pancreas, gastrointestinal tract and lung [7, 8]. In 2011, the multiple receptor tyrosine kinase (RTK) inhibitor sunitinib was approved by the FDA to treat advanced, progressive, well-differentiated pancreatic NETs [7]. The mTOR signalling pathway (including the gene changes in PTEN, TSC2, and NF1) could regulate the cell cycle, and alterations of this pathway frequently activate and promote proliferation while suppressing apoptosis, which were observed in the NETs of the gastrointestinal tract and lung [7, 8]. Everolimus, an mTOR pathway inhibitor, was approved by the FDA to treat advanced, well-differentiated, non-functional gastrointestinal and lung NETs [7, 8]. However, for the time being, information on the gene changes of cervical grade 2 NETs remains unknown. Whether there are actionable alterations in the genes of the disease is poorly elucidated.

We aimed to discuss the histological and cytogenetic characteristics of cervical grade 2 NETs in comparison with those of the gastrointestinal tract, pancreas and the lung to discover a possible algorithm that may help clinicians in diagnosing and treating patients with these uncommon and aggressive malignancies.

## Methods

### Patient enrolment

Cases of grade 2 NETs of the cervix were screened using the keywords “cervix” and “grade 2 neuroendocrine tumor” and retrieved from the electrical records of the pathology department at Peking Union Medical College Hospital (PUMCH) from 2011 to 2018. The use of archived samples was approved by the ethics committee of the PUMCH (S-K608). Haematoxylin and eosin-stained (HE) sections were reviewed independently by two experienced pathologists (RZ and ZH). The diagnosis of grade 2 NETs of the cervix was established according to the 2014 World Health Organization (WHO) classification of tumors of female reproductive organs, which indicated that grade 2 NETs or atypical carcinoid tumors are primarily defined by the same architectural and cytological features used at other sites characterized by abundant cytoplasm, characteristic granular chromatin and visible to prominent nucleoli with a greater degree of nuclear atypia and mitotic activity, as well as rare areas of necrosis compared to grade 1 tumors. Growth patterns include nested, island, organoid, spindled or trabecular. Currently, there is no specific evidence for the formulaic use of the Ki-67 proliferation index and mitotic count for the grading of cervical neuroendocrine tumors [2]. Representative tissue blocks were retrieved, and those with insufficient materials for subsequent analysis were excluded from the study. The study protocol was approved by the Institutional Review Board of PUMCH.

### Immunohistochemistry

Four-micrometre-thick sections were cut from representative formalin-fixed, paraffin-embedded tissue blocks. After deparaffinization, the sections were subjected to a panel of markers with antibodies against the following markers: AE1/ AE3, Cg A, Syn, CD56, P16, CAM5.2, PGP9.5, ER, PR, P53, P63, P40, CK7, CK20 and Ki-67 (antibody information is detailed in Table 1). The staining was accomplished using the Dako link48 autostainer following the instructions of the manufacturer. The IHC slides were interpreted by two experienced pathologists (RZ and ZH). All markers except Ki-67 and P53 were scored as either positive or negative, while Ki67 as a nuclear marker of cells was analysed on 1000 tumor cells in the areas of the highest nuclear labelling index and expressed as a percentage of stained cells according to the literature [9]. The P53 was reported using one of the four reporting patterns: normal/wild-type, complete absence, overexpression, or cytoplasmic.

### Library Preparation and Target Region Sequencing

Ten five-micrometre-thick sections were cut from each representative FFPE tissue block. DNA was extracted from the sections using the QIAmap DNA FFPE Tissue Kit (QIAGEN, USA). Extracted DNA was then amplified using ligation-mediated PCR (LM-PCR), purified, and hybridized to the probe for enrichment. The exome sequences were efficiently enriched from 1.0 μg of genomic DNA using the Agilent liquid capture system (Agilent SureSelect Human All Exon V5) according to the manufacturer’s protocol. To get the target gene regions, we designed probes on the website of Agilent about 560 genes according the design description. First, qualified genomic DNA was randomly fragmented to an average size of 180–280 bp using the Covaris S220 sonicator. Second, the gDNA fragments were end repaired and phosphorylated, followed by A-tailing and ligation at the 3′ ends with paired-end adaptors (Illumina) with a single “T”-base overhang and purification using AMPure SPRI beads from Agencourt. Then, the size distribution and concentration of the libraries were respectively determined using the Agilent 2100 Bioanalyzer and qualified using real-time PCR (2 nm). Both non-captured and captured LM-PCR products were subjected to real-time PCR. Each captured DNA library was then loaded on a Hiseq 4000 platform for paired-end 150-bp reads, and high-throughput sequencing was performed for each captured library independently. Valid sequencing data were mapped to the reference human genome (UCSC hg19) using Burrows-Wheeler Aligner (BWA) software to get the original mapping results stored in BAM format [10]. Then, SAM tools [11] and Picard (http://broadinstitute.github.io/picard/) were used to sort BAM files and perform duplicate marking, local realignment, and base quality recalibration to generate the final BAM file for computing the sequence coverage and depth. Single nucleotide variations (SNVs) and small insertions and deletions (InDels) were called using GATK and SAM tools, respectively [12]. Polymorphisms of the SNVs and InDels referenced in the 1000 Genomes Project [13] and the Exome Aggregation Consortium (ExAC) [14] with a minor allele frequency over 1% were removed. Subsequently, VCF (Variant Call Format) was annotated by ANNOVAR [15].

## Results

### Clinical characteristics

The ages of the patients ranged from 46 to 69 years with an average age of 54 years. All patients experienced vaginal bleeding. According to the International Federation of Gynecology and Obstetrics (FIGO), all NET cases were in stage IIb. All patients were treated with surgery. Case 1 was treated with chemoradiotherapy before and after surgery, while cases 2 and 3 were treated before and after surgery with chemotherapy. The follow-up time of case 1 was 27 months after the operation, however, she died because of metastases to the liver and bone. The follow-up time of case 2 and case 3 was 51 months and 74 months after the operation, respectively, and both patients survived without recurrence or metastasis.

### Pathological features

**Fig. 1:**
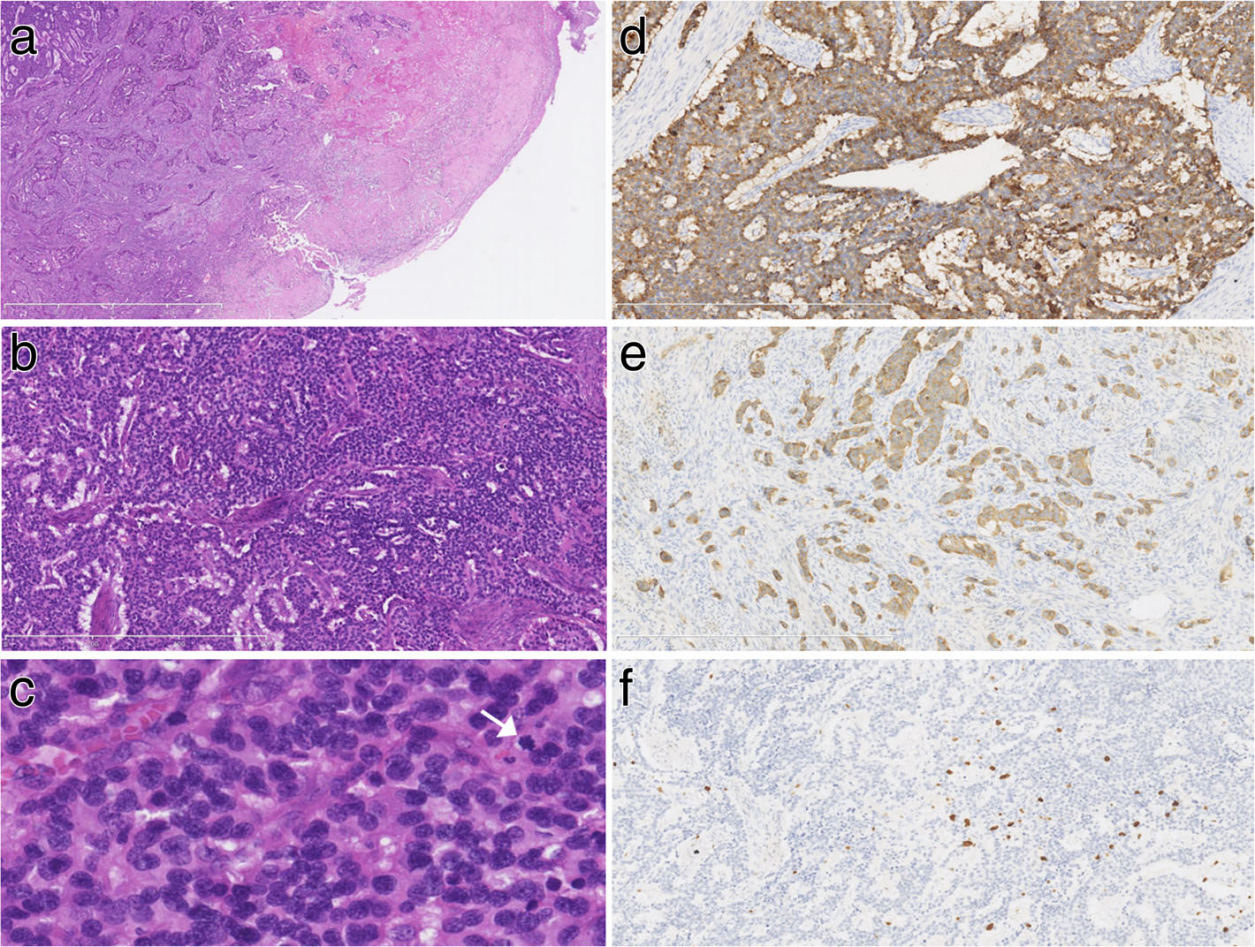
(**a**) The tumor infiltrated into the deep muscular layers. (HE; original magnification, × 25) (**b**) The tumor took the form of nest-like, trabecular and rosette-like structures. (HE; original magnification, × 100) (**c**) The tumor cells had relatively abundant eosinophilic cytoplasm and nuclei containing dense granular chromatin. The mitotic figure was observed. (HE; original magnification, × 400) (**d**) Immunohistochemically, the tumor cells were positive for Cg A. (**e**) Immunohistochemically, the tumor cells were positive for Syn. (**f**) The index of Ki-67 was 5%. (d-f immunohistochemical staining; original magnification, × 100)

The tumor size of two patients ranged from 3 cm to 5 cm, and there was no obvious lesion in the uterine cervix of the third patient. The microscopic examination revealed that the tumors of all 3 cases focally infiltrated into the deep muscular layers (Fig. 1a) with nest-like, trabecular, sheet-like, “single file” strands or rosette-like structures (Fig. 1b). Lympho-vascular invasion was observed in all cases. The tumor cells showed relatively abundant eosinophilic cytoplasm and nuclei containing dense granular chromatin. More pleomorphic and inconspicuous nucleoli were observed in patient 1 than in patient 2 and patient 3. The mitotic activity (0 to 2 mitotic figures in every 10 high-power fields) was low within the tumors in patient 1 and patient 2, and there were no necrotic foci; while an increase in mitotic activity (5 mitotic figures in every 10 high-power fields) was observed, and necrotic foci were showed in patient 1 (Fig. 1c). The immunohistochemical study showed that the tumor cells were immunoreactive for AE1/AE3, Cg A (Fig. 1d), Syn (Fig. 1e), CD56, P16, CAM5.2, and PGP9.5 and negative for ER, PR, P63, P40, CK7, and CK20. The P53 expression showed as normal/wild-type, and the Ki-67 proliferation index was 7% (Fig. 1f), 5% and 2% for the first, second and third patient, respectively. According to the morphologic findings and immunohistochemical expression profile, the diagnosis of grade 2 NETs of the uterine cervix were confirmed. (The results of the immunohistochemical analysis with the listed antibodies are provided in Table 1).

**Table 1:** Clones, Dilutions, Sources and Results of Antibodies Used in the Immunohistochemical Panel

Antibodies to	Clone	Source	Dilution	Grade 2 neuroendocrine tumors
AE1/AE3	AE1/AE3	Dako	Prediluted	+
CK20	EP23	ZSJQ-BIO, Beijing, China	Prediluted	–
CgA	EP38	ZSJQ-BIO, Beijing, China	Prediluted	+
Syn	27G12	Leica	Prediluted	+
CD56	1B6	Leica	Prediluted	+
P63	UMAB4	ZSJQ-BIO, Beijing, China	Prediluted	–
P40	ZR-8	ZSJQ-BIO, Beijing, China	Prediluted	–
CK7	OV-TL 12/30	ZSJQ-BIO, Beijing, China	Prediluted	–
P16	E6H4	Roche	Prediluted	+
Ki-67	MIB-1	ZSJQ-BIO, Beijing, China	Prediluted	Label index 2–7%
PGP9.5	13C4/I3C4	Abcam	Prediluted	+
ER	SP1	Roche	Prediluted	–
PR	SP2	Roche	Prediluted	–
P53	DO-7	MX-BIO, Fuzhou, China	Prediluted	–
CAM5.2	CAM5.2	ZSJQ-BIO, Beijing, China	Prediluted	+

*CgA* Chromogranin A;Syn, synaptophysin, ZSJQ-BIO, Zhong Shan Jin Qiao Biology; MX-BIO, Mai Xin Biology; +, positive; −, negative

### Molecular abnormalities

**Fig. 2:**
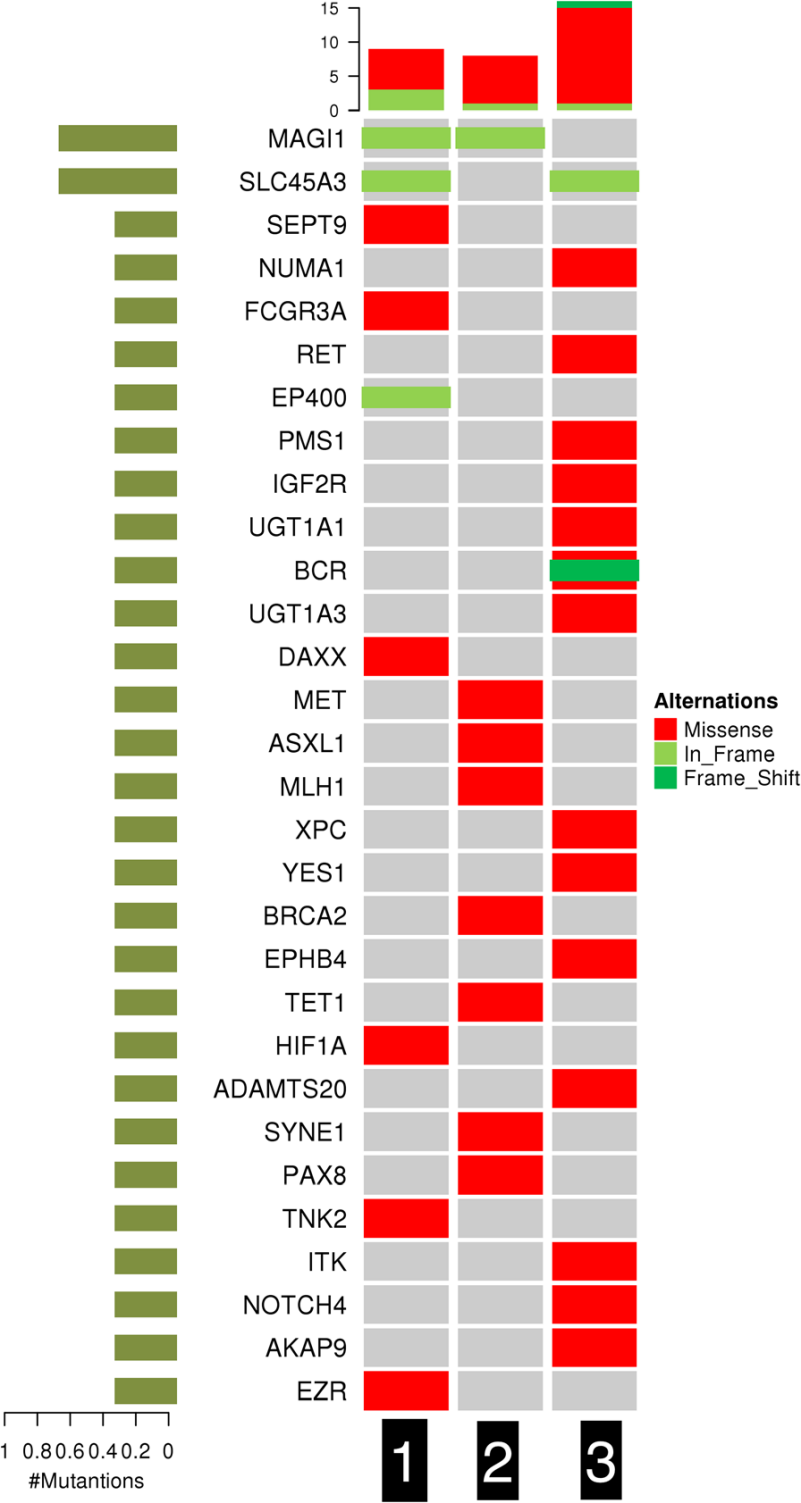
The non-frameshift insertion of MAGI1 was observed in cases 1 and 2, and the non-frameshift insertion of SLC45A3 was showed in cases 1 and 3. The missense mutation was detected, and so was the non-frameshift deletion and the frameshift insertion in the cases

A total of 560 genes were sequenced by next-generation sequencing for each patient, and nonsynonymous somatic mutations were identified in the three cases. We detected the missense of FCGR3A, STK36, TNK2, DAXX, EZR, HIF1A, and SEPT9 in case 1; the missense of PMS1, UGT1A3, XPC, ITK, NOTCH4, IGF2R, AKAP9, EPHB4, RET, KAT6B, NUMA1, ADAMTS20, ADAMTS20, YES1, and BCR in case 2; and the missense of PDE4DIP, PAX8, MLH1, SDHA, MET, TET1, BRCA2, and ASXL1 in case 3 (Table 2). Case 1 had the non-frameshift insertions of both SLC45A3 and MAGI1, while case 2 had a non-frameshift insertion of MAGI1 and case 3 had a non-frameshift insertion of SLC45A3. Case 1 also harbored an EP400 nonframeshift mutation, while case 3 had a frameshift mutation of BCR. (Fig. 2). The tumor mutation burden (TMB) for the three patients was 5.00/Mb, 3.89/Mb, and 8.89/Mb, respectively. The detailed mutational profile is documented in Table 2 and presented in Fig. 2.

**Table 2:** The genetic changes of the 3 patients

sample	genename	Achange	Exonicfuction	
case1	FCGR3A	missense	p.L66R	
case1	STK36	missense	p.R1112Q	
case1	TNK2	missense	p.R877H	
case1	DAXX	missense	p.A486G	
case1	EZR	missense	p.N6S	
case1	HIF1A	missense	p.A588T	
case1	SEPT9	missense	p.P127L	
case1	SLC45A3	nonframeshift deletion	p.222_234del	
case1	MAGI1	nonframeshift insertion	p.T422delinsQT	
case1	EP400	nonframeshift deletion	p.2728_2728del	
case2	PDE4DIP	missense	p.A272T	
case2	PAX8	missense	p.P263L	
case2	MLH1	missense	p.R217C	
case2	SDHA	missense	p.A5333V	
case2	MET	missense	p.N375S	
case2	TET1	missense	p.S487 L	
case2	BRCA2	missense	p.I3412V	
case2	ASXL1	missense	p.G652S	
case2	MAGI1	nonframeshift insertion	p.T422delinsQT	
case3	PMS1	missense	p.R883H	
case3	UGT1A3	missense	p.R45W	
case3	UGT1A5,UGT1A8,UGT1A10,UGT1A6,UGT1A7,UGT1A3,UGT1A1,UGT1A9,UGT1A4	missense	p.P364L	
case3	XPC	missense	p.L48F	
case3	ITK	missense	p.L582 M	
case3	NOTCH4	missense	p.G534S	
case3	IGF2R	missense	p.N2348S	
case3	AKAP9	missense	p.R3712Q	
case3	EPHB4	missense	p.A371V	
case3	RET	missense	p.T278 N	
case3	KAT6B	missense	p.V1499I	
case3	NUMA1	missense	p.L344 V	
case3	ADAMTS20	missense	p.S1273F	
case3	ADAMTS20	missense	p.R1000H	
case3	YES1	missense	p.I198V	
case3	BCR	missense	p.D1106N	
case3	SLC45A3	nonframeshift deletion	p.222_234del	
case3	BCR	frameshift insertion	p.S1092 fs

## Discussion

The 2014 World Health Organization (WHO) Classification of cervical NETs has been updated to adopt the terms of low-grade NETs (including grade 1 and grade 2, encompassing what were previously called carcinoids and atypical carcinoids) and high-grade neuroendocrine carcinoma (encompassing what were previously called small cell carcinoma and large cell carcinoma) [2]. Small cell carcinoma of the cervix represents the most common cervical NET, followed by cervical large cell neuroendocrine carcinoma and low-grade NETs [16, 17]. Grade 2 or atypical carcinoid tumors represent a proportion of the rarest entity of cervical NETs. A PubMed search revealed that only 17 grade 2 NETs had been reported [3–6]. In this study, we present a series of three cervical grade 2 NETs with their clinicopathological and molecular features.

The morphology of our cases did not fall beyond the description of existing studies. Grade 2 neuroendocrine tumors are well-differentiated. The histo-morphological features of these tumors are similar to the features of lung and gastrointestinal tract neuroendocrine neoplasms. Grade 2 neuroendocrine tumors take the form of nested organoid, columnar, insular or trabecular patterns. The tumor cells are epithelioid or spindled and feature relatively abundant eosinophilic cytoplasm and nuclei with dense granular chromatin, and at times, pleomorphic and inconspicuous nucleoli [2]. Necrosis can be discovered in focal areas, and the mitotic count is 2–10 mitotic figures per 10 high-power fields. The tumors of our 3 cases also showed nest-like, trabecular, sheet-like, “single file” strands or rosette-like structures, and lympho-vascular invasion was also observed. A total of 2 to 5 mitotic figures in every 10 high-power fields was observed within the tumors of our 3 cases, and necrotic foci were only observed in case 1 who died because of metastases to the liver and bone.

Grade 2 NETs of the uterine cervix are rare and probably underdiagnosed or misdiagnosed. Immunohistochemistry is useful for detecting neuroendocrine differentiation. Immunohistochemically, grade 2 NETs are diffuse and positive for synaptophysin (Syn), chromogranin A (Cg A) and CD56, and the Ki-67 proliferation index indicates the malignant characteristics of neuroendocrine tumors. In our cases, neuroendocrine markers including Syn, Cg A and CD56 were positive, and the Ki-67 proliferation index was between 2% and 7%, which was similar to the grade 2 NETs (atypical carcinoids) of the cervix reported previously [3–6]. We also stained for P40, P63, and CK7 markers for the differential diagnosis of poorly differentiated squamous cell carcinoma and adenocarcinoma, as the histology could be confusing in the case of grade 2 NETs. The patients in our study had a thorough check-up using MRT, CT, and ultrasound. No tumors were found in other parts of the body. Therefore, the detailed and rigorous clinicopathological analysis made metastatic grade 2 NETs unlikely.

Although grade 2 NETs are exceedingly rare, what is different from the neuroendocrine neoplasms in the lung and gastrointestinal tract is that these tumors often have a highly aggressive clinical behaviour, with frequent subclinical lymphatic and haematogenous metastasis [3–6]. The recognition of them is important because they are considered to be highly aggressive tumors that usually feature lympho-vascular space invasion and lymph node involvement, even in the early stage of disease. Moreover, grade 2 tumors have the propensity for local and distant relapse. The clinical outcomes and treatment information of all cases published in PubMed were reviewed. Unlike the well-differentiated neuroendocrine tumors of the gastrointestinal tract and the lung, there are only a small number of reported cases of uterine grade 2 tumors, and these data indicated no specific recommendations for the treatment of cervical grade 2 neuroendocrine tumors; however, they pose a considerable therapeutic challenge for the gynaecologic oncologists [17].

The molecular and genetic treatment of well-differentiated NETs occurring in the pancreas, gastrointestinal tract and lung has been dramatically improved by agents targeting the multiple receptor tyrosine kinase (RTK), vascular endothelial growth factor (VEGF) or the mammalian target of rapamycin (MTOR). The RTK inhibitor sunitinib was approved for the treatment of well-differentiated pancreatic NETs in 2011, and in 2016, the MTOR inhibitor everolimus was approved for well-differentiated NETs occurring in the gastrointestinal tract and the lung [7, 8]. Other biologics such as the VEGF-A inhibitor bevacizumab have also provided promising clinical solutions [7]. To confirm whether there are similar genetic changes occurring in grade 2 NETs compared with those of the pancreas, gastrointestinal tract and lung, the molecular and genetic changes in cervical grade 2 tumors of our three cases were studied. Unfortunately, the alterations of MTOR and the RTK pathway were not observed in the 3 cases, whereas other genetic changes were observed, such as the non-frameshift insertion of MAGI1 (cases 1 and 2) and SLC45A3 (cases 1 and 3). The case 1 with the non-frameshift insertion of both MAGI1 and SLC45A3 was died 27 months after the operation because of metastases to the liver and bone, while the molecular mechanism of MAGI1 and SLC45A3 to the cervical grade 2 tumor of this case is sill unknown and need to further study. The missense mutations, the non-frameshift deletion and the frameshift insertion in one of the three cases were also detected (as shown in Fig. 7). However, the changes and significance of these genetic changes for the treatment of cervical grade 2 neuroendocrine tumors have not been reported previously. Membrane-associated guanylate kinase inverted 1 (MAGI1), as a member of the membrane-associated guanylate kinase family, was downregulated in diverse cancers and was a tumor suppressor in colorectal cancer, hepatocellular carcinoma, cervical cancer and gastric cancer in previous reports [18–22]. Christian K et al. reported that MAGI-1 is a sensitive proteolytic substrate for both the HPV-16 and HPV-18 E6 oncoproteins, and its expression is always lost in HPV-positive cervical cancer cells [22]. Their findings also suggested that E6-mediated inhibition of MAGI-1 function contributes to HPV pathology by perturbing tight junction assembly with concomitant stimulation of proliferation and inhibition of apoptosis, and the restoration of MAGI-1 expression in HPV-positive cervical tumor cells could induce cell growth arrest and apoptosis [22]. Our study showed the non-frameshift insertion of MAGI1 in two cases and the immunohistochemically positive P16 indicated HPV infection of all our 3 cases [23], while the relation of MAGI1 and the HPV E6 oncoproteins was unknown and needs to further study in our cases. A study reported that the rearrangement of ERG with SLC45A3 and the loss of SLC45A3 expression were one of the aggressive pathways of prostate cancer progression [24]. The non-frameshift insertion of SLC45A3 was shown in two of our cases, and the study of the relationship between the non-frameshift insertion of SLC45A3 and the cervical grade 2 NETs is recommended. The mutation spectrum of the three cases presented did not overlap with an earlier study on the molecular profile of cervical NETs. Deyin X et al. reported next-generation sequencing based on a 637-gene panel of small cell neuroendocrine carcinoma of the uterine cervix in 10 cases and found the mutations of the gene changes of TP53, PIK3CA, KRAS, Erbb2, c-Myc, NOTCH1, BCL6, NCOA3, PTEN, RB1, BRCA1, BRCA2, and ARID1B; genetic alterations involving the MAPK, PI3K/AKT/mTOR, and TP53/BRCA pathways were also observed in their study [25]. While We detected the mutations of FCGR3A, STK36, TNK2, DAXX, EZR, HIF1A, SEPT9, PMS1, UGT1A3, XPC, ITK, NOTCH4, IGF2R, AKAP9, EPHB4, RET, KAT6B, NUMA1, ADAMTS20, YES1, BCR, PDE4DIP, PAX8, MLH1, SDHA, MET, TET1, BRCA2, ASXL1, MAGI1, SLC45A3, EP400 based on a 560-gene panel of grade 2 NETs of the uterine cervix in 3 cases, which indicated that the cervical grade 2 NETs may be have their own unique gene alterations and regulations compared with those of cervical small cell neuroendocrine carcinoma. In-depth investigation in a larger cohort of genetic alterations could provide more useful information to guide the molecular characterization and potential individualized treatment of grade 2 cervical NETs.

## Conclusion

We presented the first mutation profile revealed by whole exome sequencing in a series of grade 2 cervical NETs along with their clinicopathological characteristics. Despite being bland looking, the disease is highly invasive by nature. The non-frameshift insertions at the MAGI1 and SLC45A3 genes were both observed in case 1 who died 27 months after the operation because of metastases to the liver and bone, while the molecular mechanism of MAGI1 and SLC45A3 to the cervical grade 2 tumors of the patients is sill unknown and need to further study. Further research using a larger sample size is warranted to better characterize the disease from the molecular perspective and help identify an individualized therapeutic approach.

## Data Availability

The database generated and analyzed during this study is included in this article and its supplementary materials. Raw data are available upon reasonable request.

## Abbreviations

CgA: Chromogranin A
HE: Hematoxylin & eosin
MAGI1: membrane-associated guanylate kinase inverted 1
MTOR: mammalian target of rapamycin
NETs: neuroendocrine tumors
PUMCH: Peking Union Medical College Hospital
RTK: multiple receptor tyrosine kinase
Syn: synaptophysin
VEGF: vascular endothelial growth factor
